# A retrospective review of measles outbreaks in Ibb Governorate, Yemen (2020–2023)

**DOI:** 10.1186/s41182-025-00776-8

**Published:** 2025-07-21

**Authors:** Wadee Abdullah Al-Shehari, Wadhah Hassan Edrees, Eglal Ahmed Qasem, Yahya Ali Al-Qadhi, Abdulrahman Abdullah Humaid, Ali Ahmed Al-Halani

**Affiliations:** 1 Department of Medical Laboratories, Faculty of Medicine and Health Sciences, Ibb University, Ibb, Yemen; 2 Department of Medical Laboratories, Faculty of Medical Sciences, Aljazeera University, Ibb, Yemen; 3 Department of Medical Laboratories, Faculty of Medicine and Health Sciences, Hajjah University, Hajjah, Yemen; 4 Department of Medical Laboratories, Faculty of Medical Sciences, Al-Razi University, Sana’a, Yemen; 5 Department of Medical Laboratories, Faculty of Medicine and Health Sciences, Sana’a University, Sana’a, Yemen; 6 Biological Sciences Department/ Microbiology Division, Faculty of Sciences, Sana’a University, Sana’a, Yemen; 7 Department of Medical Laboratories, Faculty of Medical Sciences, University of Modern Science, Sana’a, Yemen; 8 Department of Medical Microbiology, Faculty of Applied Sciences, Hajjah University, Hajjah, Yemen; 9 Department of Health Administration, Al-Manar College for Science and Technology, Hajjah, Yemen

**Keywords:** Child, Disease outbreaks, Epidemiological trends, Incidence, Measles, Retrospective studies, Vaccination coverage, Yemen

## Abstract

**Background:**

Measles, a highly infectious disease that can lead to serious health problems and even death, has recently recurred worldwide despite the measures taken, with Yemen experiencing the most outbreaks among countries worldwide. There is a scarcity of current information regarding the measles epidemic in the Ibb Governorate of Yemen. Consequently, this retrospective analysis aimed to ascertain the pattern of measles outbreaks in Ibb Governorate, Yemen, during the 4-year period from 2020 to 2023.

**Methods:**

The secondary measles data contained in the database of the Health and Environment Office at Ibb Governorate between 2020 and 2023 were used for this retrospective analysis.

**Results:**

Of the 3,898 suspected measles cases, the majority of cases were recorded among males (53.2%), in the 0–4 age group (65.5%), in autumn (31.6%), and in 2023 (52.5%). The cumulative incidence rate of measles cases was 12.6 cases per 10,000 population, with the highest rates in males (13.7 cases) and the age group of 0–4 years (57.9 cases). The incidence rate has been increasing between 2020 and 2023, from 0.7 to 6.8 cases per 10,000 people. Furthermore, the overall fatality rate for measles cases was 0.87% and was significantly higher in children aged 0–4 years. The districts of As Saddah (44.7 cases) and Yarim (30.6 cases) recorded the highest incidences of measles. Most of the people who had measles had a rash (3,898; 100%), fever (3,887; 99.7%), cough (3,269; 83.9%), and runny nose (2,763; 70.9%). Additionally, 60.8% (2,370) of the cases were among unvaccinated individuals.

**Conclusion:**

Current findings indicate that measles cases are increasing over the years and could pose a significant threat to the population if left unaddressed. A stronger vaccination program, better healthcare infrastructure, the fight against vaccine hesitancy, and international collaboration are crucial for controlling and eliminating measles in this country.

## Introduction

Measles is caused by the measles virus, which is highly contagious and deadly to humans, causing significant morbidity and mortality globally. The measles virus belongs to the *Paramyxoviridae* family and *Morbillivirus* genus and is an enveloped, single-stranded RNA virus [1]. This virus is frequently transmitted through airborne droplets produced when an infected person breathes, sneezes, coughs, or laughs, or by contact with their nasal or throat secretions. The virus remains active on surfaces or in the air for up to two hours, making it easily transmitted between people, especially in crowded areas [2].

The measles virus can infect anyone and occurs most commonly among young children, particularly those who are unvaccinated or have been vaccinated against the virus but have not yet developed immunity. Furthermore, malnourished children or those with weakened immune systems due to other illnesses are at the highest risk of dying from measles [3]. The measles virus was a significant cause of illness and death on a global scale prior to the arrival of the measles vaccine, resulting in 15,000 to 60,000 cases of blindness annually and over two million deaths [4, 5].

The measles vaccine has significantly reduced infection and mortality rates caused by the measles virus over the past many years [6]. Measles cases have been rising rapidly between 2016 and 2019, from 132,490 to 869,770, increasing by 550% and resulting in approximately 207,500 deaths, which have not been observed since 1996 [7]. Globally, an estimated 9 million measles cases with 128,000 deaths were recorded in 2021 [8]. Moreover, the measles virus was responsible for approximately 136,000 deaths in 2022, the majority of which were in young children [3]. In 2023, an estimated 300,000 measles cases were reported, and in 2024, an estimated 664,144 suspected measles cases were reported worldwide [9].

According to international reports, Yemen ranked first among countries in the world in the measles epidemic in 2023 and 2025 [10–12]. Between 2018 and 2019, approximately 22,828 confirmed measles cases were reported in Yemen, of which 65% and 76% were recorded among children under 5 years of age and unvaccinated children, respectively [13]. Furthermore, an increase in the incidence of measles was documented between 2019 and 2021, from 42.4 to 142.3 cases per 1,000,000 individuals [14]. In 2022, the number of measles cases also rose rapidly, reaching 710 cases per million. In 2023, reports of suspected measles cases exceeded 50,000, resulting in 568 deaths [15].

Yemen has faced political, economic, and military challenges due to the ongoing armed conflict since 2015, which has led to the near-total destruction of its health system [16–18]. In addition, the restrictions and blockade imposed on Yemen have resulted in a lack of oil derivatives needed to operate water wells and provide drinking water, leading to increased food insecurity. This has significantly contributed to creating an environment conducive to the spread of infectious and non-communicable diseases across Yemen’s governorates [19–22].

Recently, a retrospective study documented 41,135 suspected measles cases between 2020 and 2023 in 13 governorates in southern Yemen [23]. To date, no studies have been conducted in Yemen's northern governorates to determine the magnitude of the measles epidemic in recent years. Ibb Governorate is one of the governorates that has not been studied regarding the spread of the measles epidemic. Consequently, this study aims to analyze the epidemiology of measles in the Ibb Governorate from 2020 to 2023 using surveillance data. The study's results may also help with targeted vaccination and prevention programs to stop the spread of measles in similar settings in the future.

## Methods

This analysis is a retrospective study conducted on secondary measles data recorded in the Health and Environment Office—Ibb Governorate over a period of four years between 2020 and 2023. Ibb Governorate has a strategic geographical location, as it is located between the southern and northern governorates. It is approximately 198 km away from the capital. It is bordered to the north by Dhamar Governorate, to the south by Taiz Governorate, to the east by Al Bayda and Al Dhale'e Governorates, and to the west by Al Hudaydah Governorate. It is considered the third largest governorate in terms of population 3,170,000 (10.8%) [24] and is characterized by a moderate climate throughout the year, with heavy rainfall.

### Data collection and analysis

This analysis used secondary measles data recorded between 2020 and 2023 in the database of the Health and Environment Office of Ibb Governorate. Epidemiological surveillance staff at the health office collected measles data from various hospitals and health centers across the governorate's districts over the past years and recorded them in an electronic database. In health facilities, suspected measles cases were identified based on clinical signs and symptoms and laboratory diagnostic tests using enzyme-linked immunosorbent assay (ELISA). Moreover, monthly data were extracted from the database into an Excel file, and the retrieved data were checked for consistency and completeness, cleaned, and sorted.

Furthermore, several variables were extracted from these databases. These included the date the disease started (epidemiological weeks, months, and years), demographic information (sex, age, and district), clinical signs and symptoms, vaccination information (unvaccinated, vaccinated, and number of doses), and the results of laboratory tests. Moreover, we methodically processed the missing data using the Statistical Package for the Social Sciences (SPSS), revealing the range and trends of the missing values in the database. For variables with non-significant missing values, the mean or median was used. For variables with significantly missing values, missing data entries were identified using multiple imputation techniques based on the observed data relationships. However, we excluded cases with excessive missing values that could not be reliably assessed. Additionally, consistency checks were performed after managing missing data to ensure the dependability and precision of the dataset.

This analysis included all complete data and all measles patients residing in Ibb Governorate during the study period. Data recorded before 2020 or after 2023, data from measles patients not residing in Ibb Governorate, unverified cases, and missing data were excluded.

The incidence rate of measles in the study region was calculated for the variables age, sex, years, and districts by dividing the number of new measles cases by the at-risk population multiplied by 10,000 [25]. Furthermore, the overall measles incidence rate and measles-associated mortality rate were calculated over the 4 years. The measles incidence rate was calculated for all variables, including sex, age group, year of infection, and district. The case fatality rate associated with measles was calculated for all variables, including sex, age group, year of infection, and district of infection.

The Statistical Package for the Social Sciences (SPSS) (Version 26, Version 24, IBM, USA) program was used to perform the statistical analysis on the obtained results. In addition, tables, figures, and texts were employed to present the subgroups of variables of this analysis and the frequencies and proportions of measles cases on various independent variables, using descriptive statistics. Furthermore, the chi-square test (χ^2^) was used to assess associations between measles infection and categorical variables and such as sex, age groups, case fatality rates, clinical features, and vaccination status. In addition, a confidence interval (95% CI) was also used to compare between categorical variables. Additionally, a probability (*P*) value of < 0.05 was recognized as the threshold for statistical significance.

## Results

Approximately 3,898 suspected measles cases were recorded in Ibb Governorate over the four-year period from 2020 to 2023. Males were more prevalent than females (53.2% versus 46.8%). By age, nearly two-thirds of cases were recorded among those aged 0–4 years (65.5%), more than a quarter were recorded among the 5–9 age group, and the lowest number of cases were recorded among the ≥ 15 age group (2.2%). Furthermore, the number of measles cases has increased from 238 (6.1%) in 2020 to 2,047 (52.5%) in 2023, as listed in Table 1.

**Table 1:** Sociodemographic parameters of suspected measles cases

Variables		No	(%)
Sex	Male	2074	53.2
	Female	1824	46.8
Age groups	0–4	2555	65.5
	5–9	1018	26.1
	10–14	241	6.2
	≥ 15	84	2.2
Years of onset	2020	238	6.1
	2021	447	11.5
	2022	1166	29.9
	2023	2047	52.5

### Trend of measles cases by epidemiological weeks, seasons, and months

**Fig. 1:**
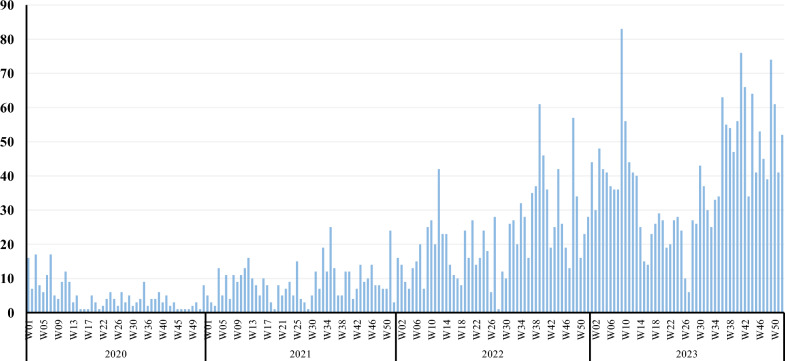
Trend of measles burden in the governorate of Ibb by epidemiological weeks from 2020 to 2023

Week 3 and Week 7 (17 cases each) recorded the highest number of measles cases in 2020, while Week 35 (25 cases) recorded the highest number of measles cases in 2021, Week 39 (61 cases) recorded the highest number of cases in 2022, and Week 9 (83 cases) recorded the highest number of measles cases in 2023, as shown in Fig. 1.

**Fig. 2:**
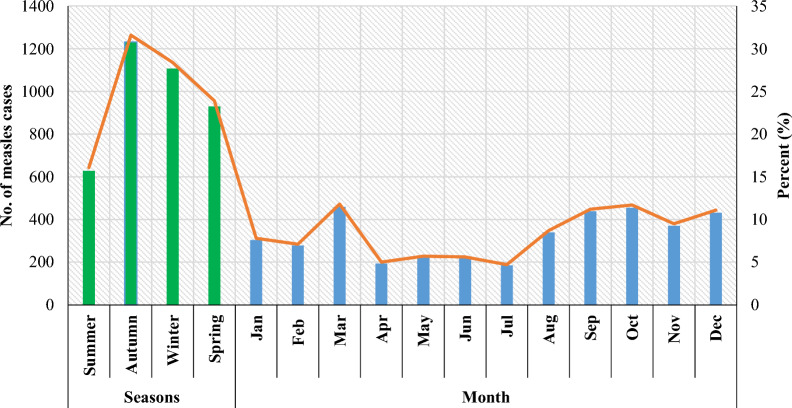
Cumulative frequency of suspected measles burden by seasons and month

These results revealed that the highest cases of measles were noted in the fall, at 1,232 (31.6%), and the lowest cases were recorded in the summer, at 626 (16.1%), with statistically significant differences (*P* < 0.05). Furthermore, March (11.8%), October (11.7%), September (11.2%), and December (11.1%) recorded the highest rate of measles cases compared to the other months, with significant differences (*P* < 0.05), as shown in Fig. 2.

### Incidence rate per 10,000 population

**Fig. 3:**
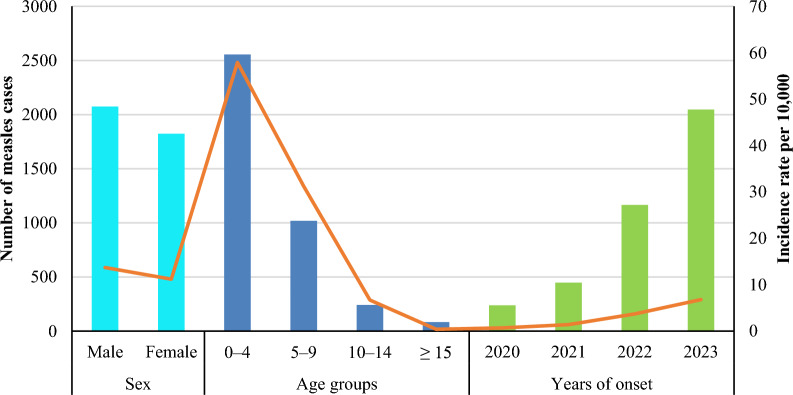
The incidence rate of measles burden by demographic variables

The current results revealed that the cumulative incidence proportion of measles cases was 12.6 cases per 10,000 populations over the four years. Furthermore, the highest incidence rate was found among males, with 13.7 cases per 10,000 populations, compared to 11.2 cases among females, with no statistically significant differences (*χ*^2^ = 0.360; 95% CI 1.24–1.66; *P* = 0.549). It was noted that the 0–4 age group recorded more than half of the incidence rate (57.9 cases per 10,000 individuals), followed by the 5–9 age group, which recorded nearly a third of the incidence rate at 31.2 cases per 10,000 people, and the lowest incidence rate was recorded among those aged 15 years or older, at 0.4 cases per 10,000 people, with statistically significant differences (*χ*^2^ = 40.227; 95% CI 1.34–1.61; *P* = 0.000). According to the years of infection, it was noted that the trend of the incidence rate increased between 2020 and 2023, between 0.7 and 6.8 cases per 10,000 people (*χ*^2^ = 7.615; 95% CI 2.75–3.88; *P* = 0.055), by nearly nine times (Fig. 3).

**Fig. 4:**
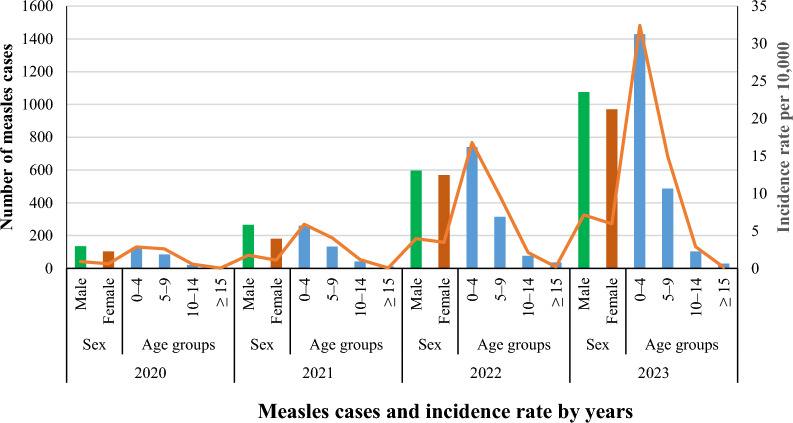
The annual incidence rate of measles burden cases by sex and age group

Figure 4 shows that the trend in the incidence rate of measles among males increased from 0.89 cases per 10,000 populations in 2020 to 7.12 cases in 2023. Regarding the age group, it was noted that the trend of measles incidence rates increased significantly among the 0–4 age group, from 2.86 cases per 10,000 populations in 2020 to 32.43 cases in 2023. Furthermore, the trend of measles incidence rates increased among the 5–9 age group, from 2.60 cases per 10,000 populations in 2020 to 14.88 cases in 2023.

**Fig. 5:**
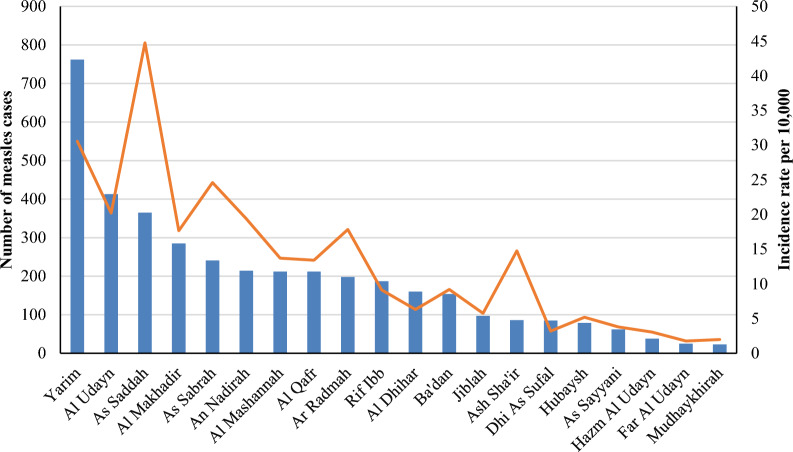
Incidence rate of measles burden by districts in Ibb governorate

Figure 5 shows that As Saddah District recorded the highest rate of measles incidence with 44.7 cases per 10,000 population, followed by Yarim District with 30.6 cases per 10,000 population, As Sabrah with 24.6 cases per 10,000 population, and Al Udayn with 20.2 cases. The Mudhaykhirah and Far Al Udayn Districts, respectively, had the lowest rate of measles incidence, with 1.9 cases and 1.8 cases per 10,000 populations.

### Case fatality rate of measles burden

**Fig. 6:**
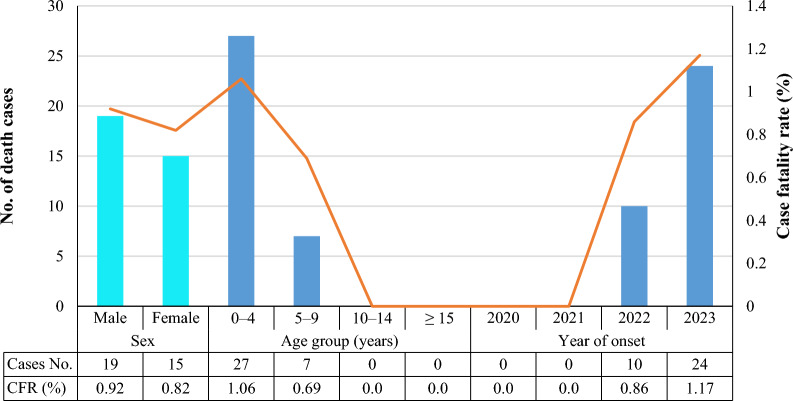
The case fatality rate of measles infection between 2020 and 2023 in Ibb governorates

Ibb Governorate recorded an overall measles case fatality rate of 0.87% (34/3898 cases) during the study period. Moreover, males (19; 0.92%) had a higher case fatality rate than females (15; 0.82%), with no statistically significant differences (*χ*^2^ = 0.471; 95% CI 1.45–1.47; *P* = 0.493). Regarding age groups, the case fatality rate was found only in children aged 0–4 years with 27 deaths (1.06%) and in those aged 5–9 years with seven deaths (0.69%), with a significant difference between age variables and measles (*χ*^2^ = 11.765; 95% CI 1.45–1.49; *P* = 0.001). Additionally, the case fatality rate was recorded only in 2023 and 2022 at 24 cases (1.17%) and 10 deaths (0.86%), respectively, with significant differences (*χ*^2^ = 5.765; 95% CI 3.26–3.30; *P* = 0.016), as shown in Fig. 6.

### Clinical features associated with measles burden

These results indicated that all measles patients had a skin rash (100%). Moreover, the majority of measles cases also had fever (99.7%), cough (83.9%), and rhinorrhea (70.9%), with a significant difference (*P* = 0.000). Furthermore, more than half of measles cases had conjunctivitis (64.1%), and less than a quarter of cases had lymphadenitis (24.2%), with significant differences (*P* = 0.000), as summarized in Table 2.

**Table 2:** Signs and symptoms associated with measles outbreak

Variables	Response	No. (%)	χ^2^	*P* value
Rash	Yes	3898 (100)	NA	NA
	No	0 (0.0)		
Fever	Yes	3887 (99.7)	3854.12	0.000
	No	11 (0.3)		
Cough	Yes	3269 (83.9)	1787.99	0.000
	No	629 (16.1)		
Runny nose	Yes	2763 (70.9)	679.93	0.000
	No	1135 (29.1)		
Conjunctivitis	Yes	2500 (64.1)	311.54	0.000
	No	1398 (35.9)		
Lymphadenitis	Yes	942 (24.2)	1040.58	0.000
	No	2956 (75.8)		

*χ*^2^ = Chi-square; *P* = Probability value < 0.05 (significant)

### Vaccination status

Of the 3,898 suspected measles cases, 2,370 (60.8%) were found to be unvaccinated, while 360 (9.2%), 773 (19.8%), and 395 (10.1%), respectively, were found to be among those who had received one dose, two doses, and three or more doses of the vaccine. Vaccination status data were verified by the immunization card. Furthermore, more unvaccinated cases were found among females (62.7%) than among males (59.2%), with no significant differences (*P* = 0.082). Additionally, the age group ≥ 15 years had the highest percentage of unvaccinated cases (70.2%), followed by the age group 0–4 years (66.0%), with a significant difference (*P* = 0.000). The majority of unvaccinated cases were found in 2023 (72.3%) and 2022 (59.0%), with significant differences (*P* = 0.000), as shown in Table 3.

**Table 3:** Measles burden by vaccination status in Ibb governorate between 2020 and 2023

Variables		Total cases (%)	Unvaccinated No. (%)	One dose No. (%)	Two doses No. (%)	≥ 3 doses No. (%)	*P*
Sex	Male	2074 (53.2)	1227 (59.2)	210 (10.1)	419 (20.2)	218 (10.5)	0.082
	Female	1824 (46.8)	1143 (62.7)	150 (8.2)	354 (19.4)	177 (9.7)	
Age groups	0–4	2555 (65.5)	1686 (66.0)	294 (11.5)	436 (17.1)	139 (5.4)	0.000
	5–9	1018 (26.1)	509 (50.0)	51 (5.0)	261 (25.6)	197 (19.4)	
	10–14	241 (6.2)	116 (48.1)	11 (4.6)	65 (27.0)	49 (20.3)	
	≥ 15	84 (2.2)	59 (70.2)	4 (4.8)	11 (13.1)	10 (11.9)	
Years of onset	2020	238 (6.1)	55 (23.1)	19 (8.0)	53 (22.3)	111 (46.6)	0.000
	2021	447 (11.5)	146 (32.7)	87 (19.5)	129 (22.9)	85 (19.0)	
	2022	1166 (29.9)	688 (59.0)	90 (7.7)	266 (22.8)	122 (10.5)	
	2023	2047 (52.5)	1481 (72.3)	164 (8.0)	325 (15.9)	77 (3.8)	

*P* = Probability value < 0.05 (significant)

## Discussion

The measles virus is highly contagious and increasingly prevalent in poor or resource-limited countries and is a major challenge facing the global health system. According to a World Health Organization report, no country has eliminated measles [6]. To our knowledge, our study is the first to document that Ibb Governorate recorded 3,898 suspected measles cases over the four-year period from 2020 to 2023. These results are similar to those of preceding studies in Yemen, which recorded 22,828 measles cases between 2018 and 2019 [13] and 41,135 suspected cases between 2020 and 2024 [22]. Moreover, other retrospective analyses conducted in various countries recorded 9, 643 measles cases in Saudi Arabia [26], 4,241 in Southeast Ethiopia [27], 11,71 in Canada [28], 306 in Taiwan [29], 401 in South Africa [30], 11,784 in Niger [31], and 26,188 in Kenya [32]. Recent observations have indicated an increase in measles cases, with the WHO European Region reporting 60,848 cases [33].

Furthermore, armed conflict has led to a significant increase in the incidence of measles (estimate = 1.131, *p* < 0.05), according to a negative binomial model conducted on 192 countries between 2018 and 2023 [34]. Armed conflict disrupts supply chains, leads to displacement from conflict zones, deteriorates healthcare, and reduces vaccination coverage and the flight of human resources. All of these factors contribute significantly to the increase in unvaccinated children and threaten herd immunity [35]. Significant rises in measles cases have been observed in the Democratic Republic of the Congo and Somalia as a result of ongoing armed conflicts [36]. Additionally, measles cases in Syria increased from 3,193 in the decade prior to the conflict to 30,241 cases between 2015 and 2019 [37]. Therefore, there is a need to enhance cooperation among countries around the world to develop and implement effective prevention strategies aimed at eliminating measles, with a focus on the most affected areas.

Seasonal variations do not appear to be associated with measles prevalence; however, travel periods and situations where unvaccinated individuals are confined in close living quarters, such as summer camps, have been linked to an increase in cases [10]. The current results revealed that the highest measles burden was observed in autumn, consistent with recent studies conducted in Ethiopia [27] and Nigeria [38]. This contradicts a previous report from some Yemeni governorates, which documented that most cases were recorded in summer and spring [23]. The discrepancy in the results is due to the geographic and climatic variations in the Yemeni governorates. In the study area, most people engage in daily activities more frequently during milder seasons, such as fall, which may contribute to measles outbreaks. Therefore, additional initiatives are needed to raise public awareness of the risks associated with infectious organisms and implement preventive measures during the seasons of greatest human movement.

This study showed that the cumulative incidence rate in Ibb Governorate over the past four years was 12.6 cases per 10,000 inhabitants, which is lower than the 18.8 cases per 10,000 population recorded in southern Yemen [23]. Furthermore, these results are higher than those documented in reports from China [39], Canada [28], Taiwan [29], Ethiopia [40], and EU countries [41]. These variations may be attributed to the effective programs employed in some nations to prevent and control measles outbreaks. Consequently, health institutions and healthcare workers must make greater efforts to enhance cooperation and coordinate efforts to monitor, detect, and track measles cases and implement plans to prevent its further spread.

These data revealed that males had a higher rate of measles incidence than females. This is consistent with earlier reports [27, 41, 42]. The current findings documented that children aged 0–4 years reported a higher incidence rate of measles, and these outcomes are inconsistent with those of other studies in Saudi Arabia [43], Lebanon [44], Syria [37], Kenya [32], Nigeria [38], and Canada [28]. The ongoing conflict in Yemen resulted in a lack of routine measles vaccinations for over 230,000 children in 2015, according to a WHO report [45]. Therefore, future measles outbreaks can be prevented by implementing various policies, including providing MMR vaccines to all children before school entry, covering all areas with vaccination staff, focusing on the most affected areas, and proactively addressing these issues.

In terms of the years of infection, the trend of the incidence rate of measles in the Ibb governorate increased between 2020 and 2023, from 0.7 to 6.8 cases per 10,000 people, by nearly nine-fold. A recent study in Yemen observed an increase in measles cases from 0.6 to 9.5 cases per 10,000 individuals between 2020 and 2023 [23]. Additionally, Patel et al. [7] documented a global increase of 556% in measles cases between 2017 and 2019. The COVID-19 pandemic that erupted from 2020 to 2022 revealed a major challenge to the global health system: delays or interruptions in measles vaccine supplies, increased outbreaks of preventable infectious diseases, and millions of children being exposed to the risk of measles infection [8]. The development of surveillance systems that are resilient to health and geopolitical crises is extremely important for the prompt detection and response to emerging outbreaks.

Regarding districts, the As Saddah district exhibited the highest rate of measles incidence per 10,000 population with 44.7 cases, followed by Yarim (30.6 cases), As Sabrah (24.6 cases), and Al Udayn (20.2 cases). The rise in measles cases in these impacted areas could be attributed to several factors, such as population density, the state of the economy, the timing of vaccinations, and the availability or quality of the vaccine. Further studies are needed to investigate the possible reasons behind the high measles outbreak among the most affected districts and to develop urgent solutions to avoid the consequences of this type of disease.

The number of measles-related deaths continues to increase annually worldwide, rising from 95,000 to 136,000 between 2021 and 2023, a 43% increase [6]. In the current results, the overall measles case fatality rate was 0.87% in Ibb Governorate, which is lower than reported in Yemen between 1 and 9.42% [13, 23]. Moreover, this outcome is also lower than reported at 1.6% in Saudi Arabia [43], 1.8% in Kenya [32], and 1.9% in Niger [31]. To achieve the goal of measles elimination, more efforts are needed to cover all areas of immunization campaigns and motivate communities to participate in routine childhood vaccination. It is also important to establish catch-up vaccination campaigns for adults and adolescents who have missed their earlier vaccinations.

Worldwide, the most measles-related deaths occur among unvaccinated or under-vaccinated children under the age of five. The measles virus weakens the immune system, making it less able to protect itself, which explains why children are more susceptible to this virus [3]. This analysis showed that the case fatality rate was higher among young children, consistent with previous studies in Yemen [13, 23] and European Union countries [41]. Therefore, primary healthcare should implement a stronger immunization program to guarantee that all children receive two doses of the measles vaccine.

Measles immunization coverage in Yemen increased from 37% in 2015 to 45% in 2023, indicating that over 50% of children in Yemen are susceptible to measles infection [46]. These results revealed that unvaccinated individuals had a significantly higher incidence of measles than vaccinated individuals (60.8% vs. 39.2%, respectively). This finding is similar to previous reports that documented 76–82.4% of measles cases among unvaccinated people in Yemen [13, 23], 85.7% in Saudi Arabia [43], 86% in the WHO European Region [40], and 63% in Canada [28]. There may be potential reasons for the low measles vaccine coverage in the study area, such as concerns about vaccine safety or effectiveness, a shortage of vaccine supplies in all areas, poor quality or invalid vaccines, or an error in the timing of vaccination.

Furthermore, social factors such as distrust in health services and political unrest that has led to the displacement of populations from areas of armed conflict may have played an additional role in preventing vaccination. Therefore, routine vaccination coverage for targeted age groups should be increased by strengthening healthcare infrastructure and community engagement. Vaccination campaigns should be implemented as scheduled. Supplementary vaccination campaigns targeting areas with low vaccination coverage should be launched. Mobile vaccination clinics and units should be established in underserved areas, providing vaccines at appropriate locations and times.

### Strength and limitations

This comprehensive analysis of the measles epidemiological situation is the first to be conducted in Ibb Governorate, a region that has been overlooked in many previous studies. The results of this analysis will significantly contribute to bridging this critical gap and provide relevant perspectives on the actual measles situation aimed at devising and implementing effective programs to enhance measles control and prevention in the study areas. Although this report boasts numerous strengths, it has some limitations. This analysis used secondary data from government health offices, which might not be complete or accurate because data quality and case reporting vary from district to district. Moreover, this study relied on passive surveillance, which could lead to underreporting of cases and possibly compromise the accuracy of our findings. In addition, there may be underreporting of measles cases, which could lead to inaccurate estimates of the prevalence of this disease. Furthermore, vaccination data may be incomplete, which could potentially impact the precision of the results and the estimates of vaccination efficacy. Additionally, this analysis was unable to assess factors such as economics and environment that are associated with measles prevalence, which will add further significance to the findings.

## Conclusion

Measles continues to pose a significant obstacle to Yemen's health system, as the number of cases continues to rise annually in the absence of effective epidemic surveillance and control strategies. Moreover, the majority of cases were observed among males, children under 5 years, in autumn, and in 2023. In addition, the trend of the incidence rate of measles increased between 2020 and 2023, and most of the cases were among the unvaccinated individuals. The districts of As Saddah and Yarim are among the districts with the highest measles endemicity. Therefore, the implementation of effective preventive strategies in Yemen will require international cooperation and support to combat and eradicate measles outbreaks. Furthermore, it is essential to launch campaigns to raise public awareness of the importance of vaccines and their role in decreasing illness and death rates. Additional research is required to identify the risk factors that contribute to the escalation of the measles burden, including socioeconomic, environmental, educational, and behavioral factors. We recommend strengthening surveillance, targeting high-incidence districts for supplementary immunization, and improving vaccine cold chain systems.

## Data Availability

The datasets used and analyzed during the current analysis available from the corresponding author on reasonable request.

## Abbreviations

CDC: Centers for disease control and prevention
CFR: Case fatality rate
MMR: Measles, mumps, and rubella
WHO: World Health Organization
*P*: Probability value
χ^2^: Chi-square test (*χ*^2^)
